# Bifunctional Europium for *Operando* Catalyst Thermometry in an Exothermic Chemical Reaction

**DOI:** 10.1002/anie.202211991

**Published:** 2022-11-24

**Authors:** Bas J. P. Terlingen, Tjom Arens, Thomas P. van Swieten, Freddy T. Rabouw, P. Tim Prins, Michiel M. de Beer, Andries Meijerink, Mathieu P. Ahr, Eline M. Hutter, Coert E. J. van Lare, Bert M. Weckhuysen

**Affiliations:** ^1^ Debye Institute for Nanomaterials Science and Institute for Sustainable and Circular Chemistry Department of Chemistry Utrecht University Universiteitsweg 99 3584 CG Utrecht The Netherlands; ^2^ Nobian Zutphenseweg 10 7418 AJ Deventer The Netherlands; ^3^ KLK Kolb Specialties Langestraat 137 7491 AE Delden The Netherlands

**Keywords:** Europium, Heterogeneous Catalysis, Methane, *Operando* Methods, Thermometry

## Abstract

Often the reactor or the reaction medium temperature is reported in the field of heterogeneous catalysis, even though it could vary significantly from the reactive catalyst temperature. The influence of the catalyst temperature on the catalytic performance and vice versa is therefore not always accurately known. We here apply EuOCl as both solid catalyst and thermometer, allowing for *operando* temperature determination. The interplay between reaction conditions and the catalyst temperature dynamics is studied. A maximum temperature difference between the catalyst and oven of +16 °C was observed due to the exothermicity of the methane oxychlorination reaction. Heat dissipation by radiation appears dominating compared to convection in this set‐up, explaining the observed uniform catalyst bed temperature. Application of *operando* catalyst thermometry could provide a deeper mechanistic understanding of catalyst performances and allow for safer process operation in chemical industries.

## Graphical abstract

Temperature determines the reaction kinetics, thermodynamics and catalyst stability. However, very limited information is known about the local catalyst temperature. Here, *operando* thermometry over bifunctional Eu^3+^ is performed to study the interplay between reaction conditions and the catalyst temperature dynamics in the exothermic methane oxychlorination reaction.

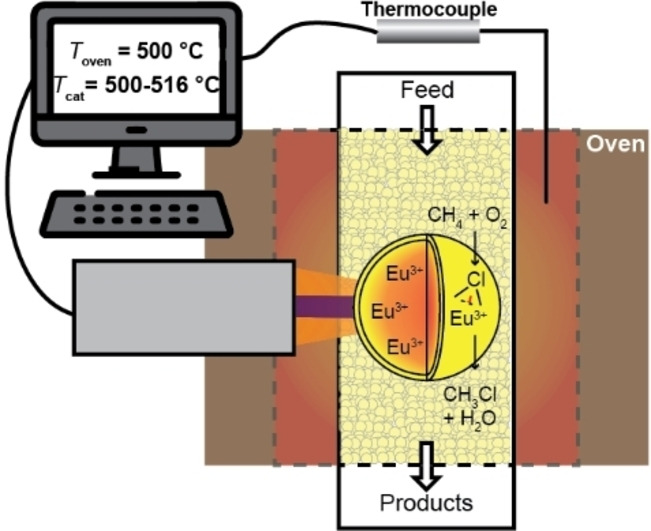

Temperature is arguably the most important parameter in catalysis as it dominates the reaction kinetics, thermodynamics and stability of the catalyst.[^1^, ^2^] These phenomena determine the overall feasibility of a chemical process. Almost all publications in the field consider only the temperature of the reactor or reaction medium, thereby assuming that the catalyst bodies have the same temperature. The influence of the reaction thermodynamics and kinetics is not considered in most cases, undermining the spatiotemporal temperature changes of the catalyst bodies under reaction conditions. It is somewhat surprising that, after more than a century of heterogeneous catalysis research, very limited information is available on the local temperature of the catalyst bodies under true working conditions further emphasizing the need and importance of *operando* spectroscopy research.[^3^, ^4^, ^5^, ^6^, ^7^, ^8^, ^9^, ^10^, ^11^, ^12^, ^13^]

In the handful of articles published on o*perando* catalyst thermometry currently available, large discrepancies between the set reactor temperature and the catalyst temperature were reported.[^7^, ^8^] Previous research evidenced that local temperature was highly influenced by the reaction mixture fed in the reactor, inducing heat generation (+80 °C from set point)^[7]^ or heat removal (−65 °C from set point).^[8]^ These results stress the importance of monitoring the local temperature as precise control is crucial for stable, safe, and efficient catalytic conversion processes.[^14^, ^15^, ^16^, ^17^] *Operando* determination of the catalyst temperature is especially interesting in exothermic reactions. As a case study, we have chosen in this work the methane oxychlorination (MOC) reaction, which has to potential to see commercialization, but operating this potentially hazardous reaction is far from trivial.[^18^, ^19^, ^20^, ^21^, ^22^, ^23^, ^24^, ^25^] The reaction CH_4_+HCl+1/2 O_2_→CH_3_Cl+H_2_O is highly exothermic, with a reaction enthalpy of Δ*H* ^0^_773K_=−157.6 kJ mol^−1^, operated at >400 °C, the reaction feed/products mixtures are corrosive, potentially explosive, and toxic.[^26^, ^27^] Process safety is thus a top priority for conducting this chemical reaction.

The application of *operando* catalyst thermometry in the methane oxychlorination reaction enables real‐time control over the catalyst performance and process safety. A hazardous thermal runaway can be identified in an early stage, without the temperature detection delay from which traditional temperature measurement techniques suffer.[^28^, ^29^, ^30^] EuOCl is a unique catalyst for methane oxychlorination, because it is not only catalytically active, but also shows temperature‐sensitive luminescence. In contrast to the method applied in literature, where typically thermometric particles are added to the catalyst, we can ensure that the temperature of the catalyst is measured.[^7^, ^8^] Furthermore, this eliminates the potential influence of the thermometric particles on the catalytic activity. Thus, this bifunctionality enables local temperature measurements at the active catalytic site without influencing the catalysis. The bifunctionality concept and the visualization of the experimental set‐up is depicted in Scheme 1. The spot size of the laser is in the millimetre range, measuring the temperature of numerous catalyst bodies (125–425 μm sieve fraction) that are agglomerates of individual EuOCl catalyst particles (<500 nm).^[27]^ Hence, an average temperature of numerous catalyst particles is obtained. At the surface of an individual catalyst particle, the methane oxychlorination reaction proceeds and heat is generated due to the exothermicity of the possible reactions (see Supporting Information section 2). Under reaction conditions, Eu^3+^ is excited with a 375 nm laser, penetrating the surface of the individual catalyst particle. This generates luminescence both at the surface and in the interior part of the catalyst, enabling temperature measurements of the entire catalyst particle. UV‐light typically has a penetration depth of <100 μm in strongly scattering media such as a bed of micrometre‐sized particles.^[31]^ Hence, penetration of the laser through the catalyst bed is not expected. We confirmed this by performing absorbance and transmittance measurements (Figure S2). The absorption at 375 nm was relatively high and less than 1 % of the incident beam could pass through the EuOCl‐filled quartz reactor.

**Scheme 1:**
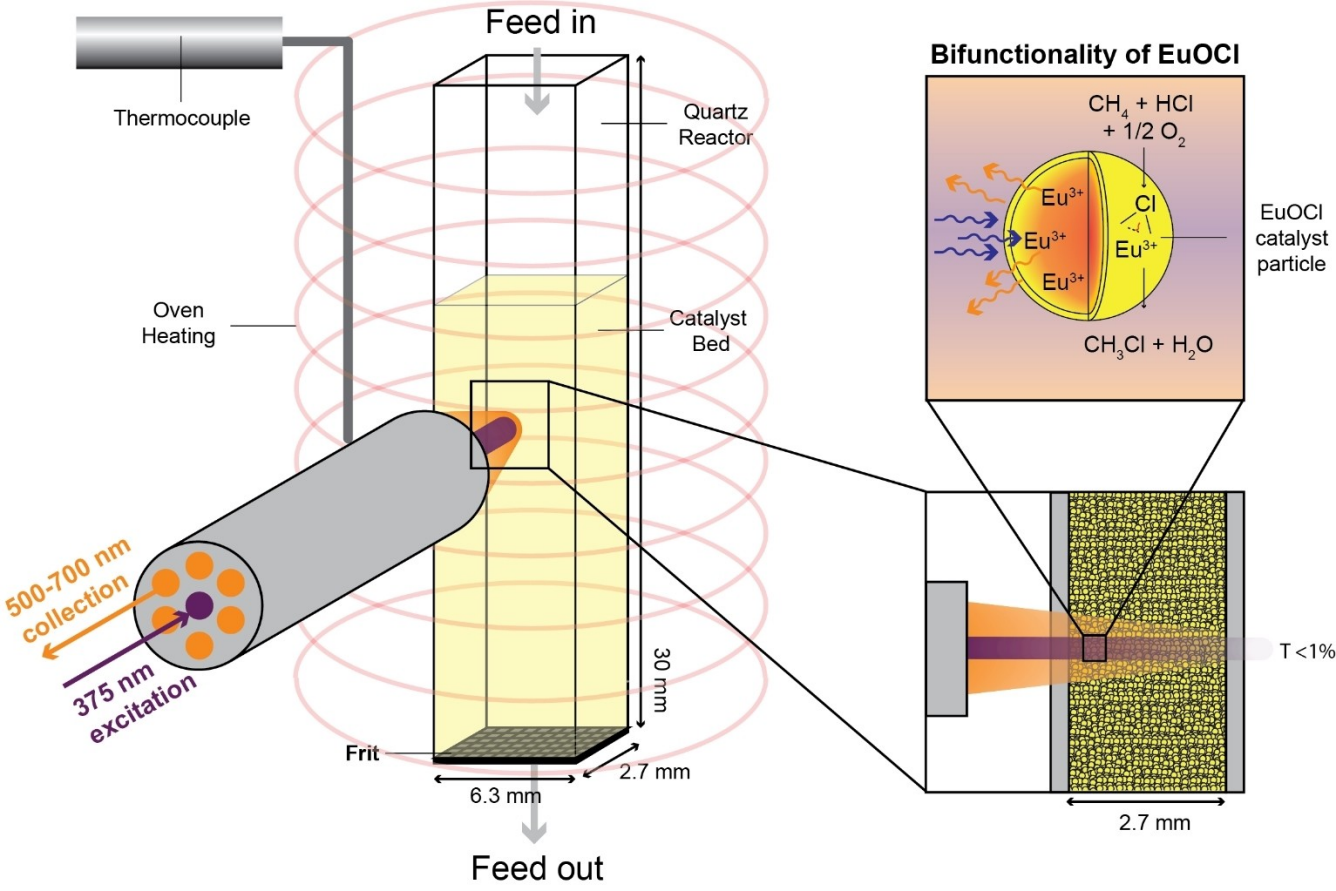
Visualization of the experimental set‐up, in which the bifunctionality of EuOCl as catalyst material and thermometer can be analyzed simultaneously. The quartz reactor is situated in the oven and the laser probe is aligned perpendicular to the quartz window. The 375 nm laser illuminates a part of the catalyst bed and the emission is collected through the same probe. The penetration of the incident beam through the filled quartz reactor is less than 1 % at 375 nm. The thermocouple is situated in the oven on the same height as the laser. The reactive feed passes the top of the catalyst bed first, and leaves through the bottom to the gas chromatograph (GC).

The bifunctional EuOCl catalyst material was synthesized (see Supporting Information section 1 for more experimental details) in the desired EuOCl crystal phase (Figure S3A) with an irregularly shaped morphology (Figure S3B).[^25^, ^27^, ^32^] The excitation of EuOCl with 375 nm light yielded sharp emissions (Figure S4), which could all be assigned according to the energy diagram of Eu^3+^ (Figure 1A).[^33^, ^34^] The EuOCl catalyst material showed temperature‐dependent emission from 400 to 550 °C (Figure 1B). Specifically, we observe variations in the relative emission intensity from the thermally coupled ^5^D_0_ and ^5^D_1_ states, specifically the ^5^D_1_→^7^F_1‐2_ and ^5^D_0_→^7^F_2_ emissions, denoted *I*_2_ and *I*_1_, respectively (Figure 1C). The temperature‐dependent emission was calibrated in inert conditions (20 mL min^−1^ N_2_, 1 °C min^−1^) and could be described according to the Boltzmann model. This yielded an energy difference between the two thermally coupled states ΔE of 1804 cm^−1^ corresponding to the expected value of 1760 cm^−1^.^[33]^ Furthermore, the catalyst temperature as determined by the Boltzmann model (*T*_cat_) was not influenced by the gas flow (Figure S5). We therefore assume that the temperature of the feed gas is equal to *T*_oven_. Bulk chlorination of EuOCl to EuCl_3_ can occur under reaction conditions when the chlorination reaction occurs at a faster rate than the dechlorination reaction.[^26^, ^27^] This must be avoided as it will yield a faulty temperature read‐out because of two reasons. The first reason is that the emission spectrum of EuCl_3_ differs from EuOCl. Hence, if the ^5^D_1_/^5^D_0_ ratio is used for the *I*_2_/*I*_1_ ratio, it will result in a measurement error. The second reason is that the luminescence signal of EuCl_3_ is quenched at reaction temperatures, inducing a large temperature uncertainty.^[27]^ Since the luminescence of EuCl_3_ is relatively weak, we can use the emission intensity as an indication for the undesired formation of EuCl_3_. Indeed, we observed a loss of luminescence signal at temperatures below 500 °C under chlorinating conditions, which is likely caused by formation of EuCl_3_ (Figure S6). To prevent bulk chlorination and ensure accurate temperature measurements, we will carry out the experiments at temperatures at or above 500 °C.

**Figure 1:**
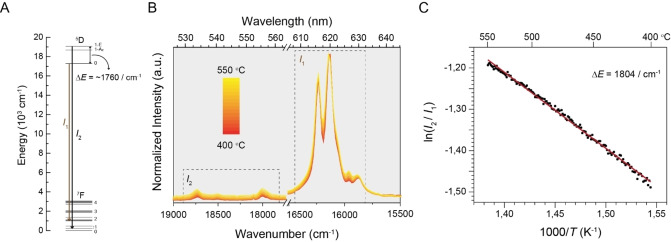
A) Energy diagram of Eu^3+^ where the energy gap (Δ*E*) between the ^5^D_1_ and ^5^D_0_ states is indicated. B) Emission spectra of EuOCl upon excitation at 375 nm from 400 (red) to 550 °C (yellow) with increments of 10 °C, where the ^5^D_1_ and ^5^D_0_ emissions are indicated by *I*_2_ and *I*_1_ respectively. C) The ratio of integrated emission intensities of the ^5^D_1_ and ^5^D_0_ states (see Supporting Information section 3.1, 3.2 and Figure S1 for details on data analysis) as a function of the inverse temperature. The straight line is a fit to the Boltzmann model, yielding a ^5^D_1_‐^5^D_0_ Δ*E* of 1804 cm^−1^.

The catalyst temperature was significantly increased due to the heat generation under MOC reaction conditions (Figure 2). A temperature ramp‐up and ramp‐down between 500 °C and 550 °C revealed a significant discrepancy between the *T*_oven_ and *T*_cat_, further denoted as Δ*T*, where catalyst heating was observed over the entire temperature range (Figure 2A). At *T*_oven_=500 °C (0–15 min), a Δ*T* of 8 °C was observed, which increased to 13 °C at *T*_oven_=550 °C (65 min, Figure 2B). When the temperature was kept at 550 °C, the Δ*T* increased further to ≈16 °C. Once *T*_oven_ was ramped down from 550 °C to 500 °C (95–145 min), the Δ*T* gradually decreased from 16 to 10 °C. The Δ*T* showed a positive correlation with the observed activity in the reaction (Figure 2C). Interestingly, an asymmetry in the Δ*T* between the ramp up and ramp down was observed. The Δ*T* was higher at the same *T*_oven_ during the ramp down compared to the ramp up, coinciding with the higher observed activity at the same *T*_oven_ (Figure 2C). As a constant gas hourly space velocity (GHSV) was used, the change in Δ*T* can be ascribed to the positive feedback between reaction rate and local temperature.

**Figure 2:**
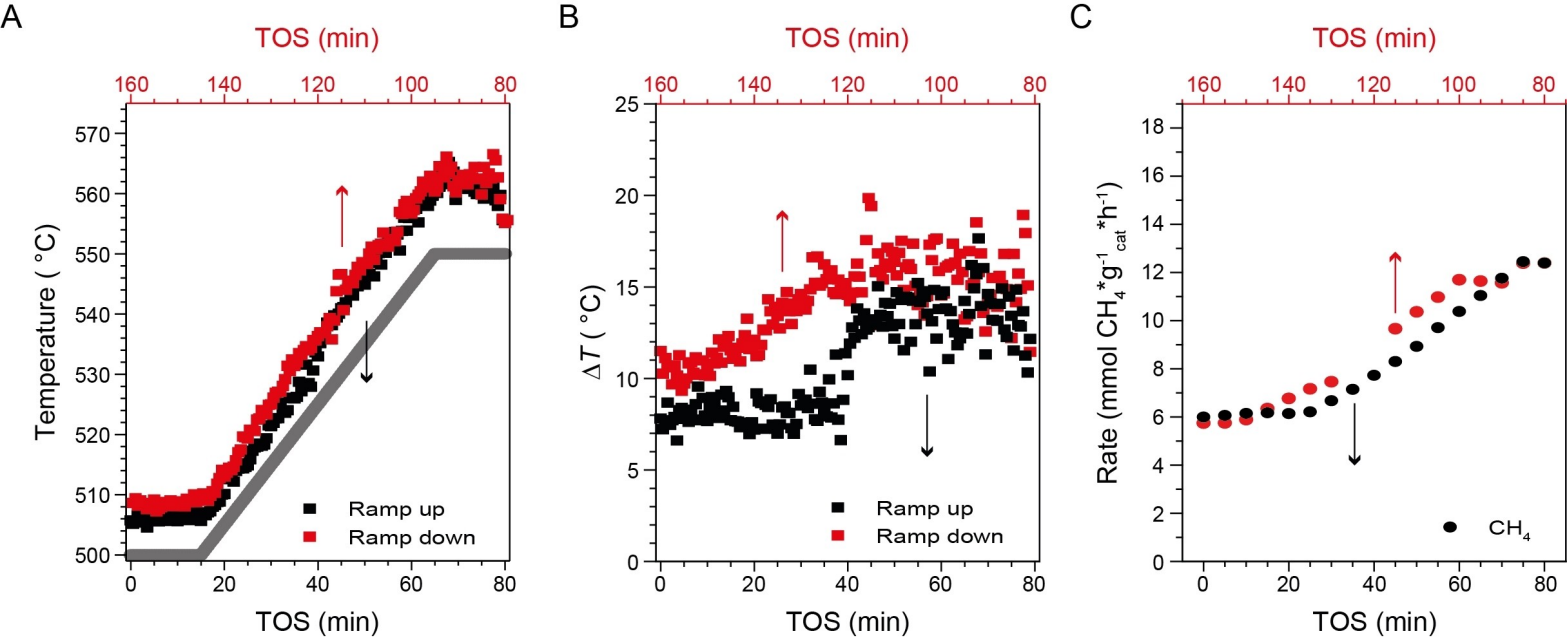
A) Temperature ramp‐up and ramp‐down between 500 °C and 550 °C where the catalyst temperature as determined by the Boltzmann model (*T*_cat_) and oven temperature (*T*_oven_) are plotted versus the time‐on‐stream (TOS). B) Δ*T* defined as the difference between *T*_cat_ and *T*_oven_ plotted versus TOS. C) Simultaneous to the temperature measurements, the activity was measured and the CH_4_ conversion rate is given. In all cases, the black data points correspond to the bottom x‐axis and the red data points correspond to the upper x‐axis. Reaction conditions: CH_4_ : HCl : O_2_ : N_2_ : He of 8 : 8 : 4 : 4 : 0 (in mL min^−1^), *T*_oven_=500–550 °C with a ramp rate of 1 °C min^−1^, *W*_cat_=500 mg.

To shed light on the interplay between catalyst cooling and heat generation, three different variations in the feed composition were investigated. The response on the activity and local temperature was analyzed. Increasing the GHSV while keeping the feed composition unaltered resulted in an increase in the Δ*T* (Figure 3A). The increase in Δ*T* can be explained by the increase in reaction rate of both O_2_ and CH_4_. The higher flow of reactive gas generated more reaction heat than was removed by the higher gas flow rate. The opposite effect was observed when the feed was diluted with inert He (Figure 3B). The increasing GHSV lowered the Δ*T* while also decreasing the reaction rate of O_2_ and CH_4_. Note that at low reaction rates, the Δ*T* was roughly 0 °C. Finally, the GHSV was kept constant while the activity was increased (Figure 3C). This was achieved by varying the HCl : He ratio while keeping a constant total flow.^[27]^ Again, the reaction rate of CH_4_ and O_2_ were positively correlated to the Δ*T*. Here, the change in Δ*T* is purely a kinetic effect as the cooling rate by convection can be regarded constant.

**Figure 3:**
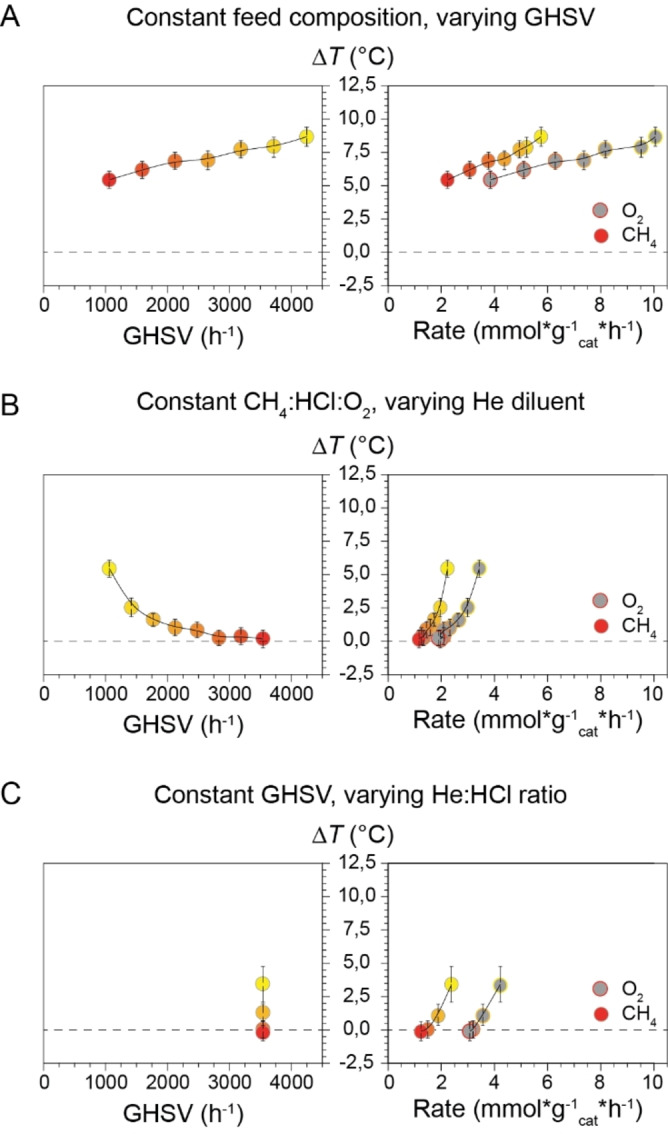
Temperature difference between the *T*_oven_ and *T*_cat_, defined as Δ*T*, plotted versus the variable parameter gas hourly space velocity (GHSV, left window) and the resulting reaction rate for CH_4_ and O_2_ (right window). A) The total flow was increased without altering the gas feed composition (CH_4_ : HCl : O_2_ : N_2_ : He 2 : 2 : 1 : 1 : 0). B) The reaction mixture was diluted with inert He (CH_4_ : HCl : O_2_ : N_2_ : He 2 : 2 : 1 : 1 : x (in mL min^−1^) where *x*=0–14 with increments of 2). C) The HCl : He ratio in the feed was altered while maintaining the same total flow (CH_4_ : HCl : O_2_ : N_2_ : He 2 : 16−*x* : 1 : 1 : *x* (in mL min^−1^) where *x*=0 (yellow), 4 (orange), 8 (dark orange), 14 (red)). See Figure S7 for more information. Reaction conditions: *T*_oven_=500 °C, *W*_cat_=500 mg, *V*_cat_=0.34 cm^3^.

Both under varying oven temperatures (Figure 2) as well as under varying reaction gas feed compositions (Figure 3), the reaction rate was positively correlated to the Δ*T*. Furthermore, an increase in the GHSV did not always result in enhanced heat removal, as the reaction rate was not correlated to the GHSV. Heat transfer calculations (Supporting Information section 3.3) evidenced that heat loss due to convection is negligible compared to radiation. Any heat generated by the reaction will quickly dissipate by radiation until an equilibrium temperature is reached. Hence, a uniform catalyst temperature over the length of the bed is expected. Our qualitative approach was able to explain the observed trends, but we were unable to describe the change in temperature quantitatively. To verify the uniformity of the catalyst bed temperature, the *T*_cat_ was determined at the top and bottom of the catalyst bed (Figure 4) under MOC conditions where the GSHV was varied. As the experimental set‐up did not allow for simultaneous temperature determination of the catalyst at the top and bottom of the bed, the temperatures were determined in a sequential manner. First the top of the catalyst was probed and subsequently the bed height with respect to the probe was altered to enable the temperature determination of the bottom part. A constant reaction rate confirmed that the catalyst activity was the same in subsequent experiments. Furthermore, the bottom part of the catalyst bed was roughly as active as the top part, since a decrease in the catalyst loading by half resulted in a reduction of the X${}_{\mathrm{CH}{}_{4}}$ by the same amount (Figure S8). Overall, we observed no significant difference in temperature between the top and the bottom of the reactor, consistent with radiation as the dominant heat dissipation mechanism.

**Figure 4:**
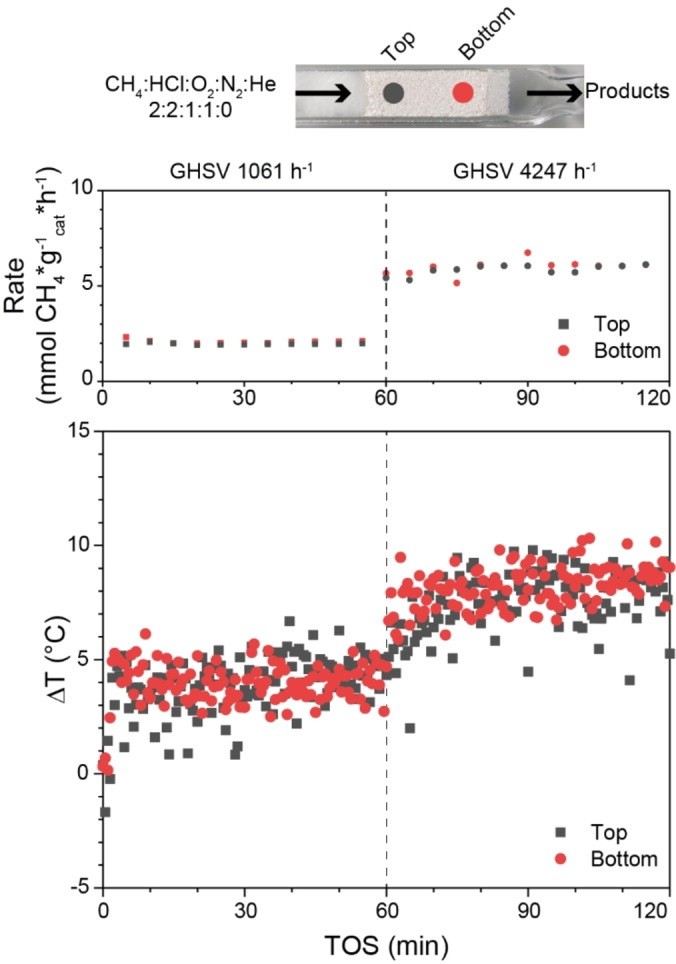
The reaction rate and the temperature difference between the *T*_oven_ and *T*_cat_, defined as Δ*T*, plotted versus the time‐on‐stream (TOS). Catalyst temperature was determined at different bed heights, separated 9 mm apart. After a stabilization period of 30 min under N_2_, the reaction mixture was introduced. The temperature of the top part and bottom part were measured sequentially. Reaction conditions: CH_4_ : HCl : O_2_ : N_2_ : He of 2 : 2 : 1 : 1 : 0 (total flow 6 or 24 mL min^−1^), *T*_oven_=500 °C, *W*_cat_=500 mg, *V*_cat_=0.34 cm^3^.

The bifunctionality of EuOCl, i.e., the catalytic activity in the MOC reaction and its temperature‐dependent luminescence, enabled the rapid, stable, and direct temperature determination of catalyst particles under reaction conditions. The apparent heat balance between heat generation and heat loss was investigated by varying the feed composition and the reaction temperature. Under various conditions we observed a higher catalyst temperature compared to the oven set temperature. This temperature difference Δ*T* correlated strongly to the reaction rate. The maximum Δ*T* observed was 16 °C. Heat‐balance calculations were able to describe the observed trends qualitatively and identified radiation as the dominant heat dissipation mechanism. This was consistent with the measured uniform catalyst bed temperature. To be able to describe the temperature increase quantitatively, a more detailed model must be constructed and more carefully controlled experiments must be conducted, which will form the basis of a follow‐up study. We anticipate that the concept of *operando* thermometry in chemical reactions can be transferred to other fields where lanthanide‐based catalysts are used. These fields include reactions where a lanthanide acts as catalytic center, e.g. reactions involving halogens,[^35^, ^36^, ^37^, ^38^, ^39^, ^40^] or promotor, e.g. reforming reactions[^41^, ^42^] and methanol synthesis^[43]^ in which La^3+^ would be (partially) substituted by Eu^3+^. Lastly, the application of thermometric support materials[^44^, ^45^, ^46^, ^47^] opens up the possibility to perform *operando* thermometry in chemical reactions where lanthanides typically do not play a role. Hence, the integrated functional material‐thermometer concept, successfully explored in this work of the MOC reaction, can be extended to a wide variety of processes and materials.

## Conflict of interest

The authors declare no conflict of interest.

## Supporting information

As a service to our authors and readers, this journal provides supporting information supplied by the authors. Such materials are peer reviewed and may be re‐organized for online delivery, but are not copy‐edited or typeset. Technical support issues arising from supporting information (other than missing files) should be addressed to the authors.

Supporting Information

## Data Availability

The data that support the findings of this study are available from the corresponding author upon reasonable request.
