# Supplementary material for: Bifunctional Europium for Operando Catalyst Thermometry in an Exothermic Chemical Reaction
Source: Angew Chem Int Ed Engl. 2022 Nov 24;61(52):e202211991. doi: 10.1002/anie.202211991 (PMC10099702; doi:10.1002/anie.202211991)
Supplement: Supplementary file 1 — Supporting Information [file ANIE-61-0-s001.pdf]

Angewandte Chemie
Eine Zeitschrift der Gesellschaft Deutscher Chemiker

Supporting Information

Bifunctional Europium for *Operando* Catalyst Thermometry in an Exothermic Chemical Reaction

*B. J. P. Terlingen, T. Arens, T. P. van Swieten, F. T. Rabouw, P. T. Prins, M. M. de Beer, A. Meijerink, M. P. Ahr, E. M. Hutter, C. E. J. van Lare, B. M. Weckhuysen**

1. Experimental Section

Catalyst Synthesis and characterization

EuOCl powders were synthesized by dissolving europium (III) chloride hydrate ($EuCl_3 \cdot xH_2O$, Alfa Aesar, >99.9%) in ethanol (absolute, VWR). Subsequently, stoichiometric amounts of ammonium hydroxide (Fisher Scientific, 25% in H_2O) were added in drop-wise fashion at room temperature. The precipitates were stirred for 1 h, centrifuged and washed with ethanol (absolute, VWR) three times, dried at 80 °C in air and calcined at 500 °C for 3 h (5 °C/min ramp rate).[1] X-ray diffraction (XRD) patterns were obtained with a Bruker-AXS D8 powder X-ray diffractometer in Bragg−Brentano geometry, using Cu $K_{\alpha1,2}$ = 1.54184 Å, operated at 40 kV. The measurements were carried out between 22 and 65 ° using a step size of 0.02 ° and a scan speed of 1 s, with a 2 mm slit for the source. Transmission electron microscopy (TEM) was performed on a FEI Tecnai 20 instrument operating at 200kV. Absorbance and transmittance measurements were performed on a PerkinElmer UV/Vis Lambda 950s with a step size of 2nm and an step time of 1s.

Operando luminescence spectroscopy was performed with an Cobolt-06-01 laser (λ = 375 nm, max laser output 75 mW) excitation source, coupled to an Avantes FCR-7UVIR400-2.5-bx-6x350-HTX reflection probe, capable of withstanding temperatures up to 550 °C. Detection was performed with an AvaSpec-ULS2048CL-EVO with a 25 mm slit (300 lines/mm). Spectra were collected with the AvaSoft 8 software. For the data processing procedure, see the Supporting information section 3.2.

Catalyst Testing

All the catalytic tests and *operando* spectroscopy characterization experiments were performed in a lab-scale continuous-flow fixed-bed reactor quartz reactor capable of withstanding the corrosive gas feed. The quartz reactor (reactor dimensions L x W x D: 30 x 6.3 x 2.7 mm, see Scheme S1) was loaded with catalyst and placed vertically in a home-made oven reaching the center. The oven has a one-sided horizontal hole where the high-temperature probe could be inserted. The oven temperature was regulated with a K-type thermocouple of which the tip reached the center of the oven at the same height as the catalyst bed and the high temperature probe. More details on the experimental set-up as well as definitions and calculations are reported elsewhere.[1] In a typical methane oxychlorination experiment, 500 mg of the catalyst material (125-425 µm sieve fraction) was loaded in a quartz reactor and heated to 500 °C with a ramp rate of 10 °C/min in 20 ml/min N_2. Subsequently, the feed was changed to the desired CH_4:HCl:O_2:N_2:He ratio specified in the caption of every figure.

2. Reaction Enthalpies

Chlorination reaction of EuOCl:

$$EuOCl + 2\,HCl \rightarrow EuCl_3 + H_2O\;(\Delta H_{773K} = -81.3\frac{kJ}{mol})$$

Dechlorination reaction of $EuCl_3$:

$$EuCl_3 + 2\,CH_4 + O_2 \rightarrow EuOCl + 2\,CH_3Cl + H_2O\;(\Delta H_{773K} = -233.9\,\frac{kJ}{mol})$$

Oxychlorination reaction of C_1:

$$2\,CH_4 + 2\,HCl + O_2 \rightarrow\; 2\,CH_3Cl + 2\,H_2O\;(\Delta H_{773K} = -315.2\frac{kJ}{mol})$$

$$2\,CH_3Cl + 2\,HCl + O_2 \rightarrow\; 2\,CH_2Cl_2 + 2\,H_2O\;(\Delta H_{773K} = -324.8\frac{kJ}{mol})$$

$$2\,CH_2Cl_2 + 2\,HCl + O_2 \rightarrow\; 2\,CHCl_3 + 2\,H_2O\;(\Delta H_{773K} = -307.5\frac{kJ}{mol})$$

$$2\,CHCl_3 + 2\,HCl + O_2 \rightarrow 2\,CCl_4 + 2\,H_2O\;(\Delta H_{773K} = -281.8\frac{kJ}{mol})$$

Catalytic destruction with H_2O:

$$CH_3Cl + H_2O \rightarrow CO + HCl + \;2\,H_2\;(\Delta H_{773K} = 132.9\frac{kJ}{mol})$$

$$CH_2Cl_2 + H_2O \rightarrow CO + 2\,HCl + H_2\;(\Delta H_{773K} = 49.2\frac{kJ}{mol})$$

$$CHCl_3 + H_2O \rightarrow CO + 3\,HCl\;(\Delta H_{773K} = -43.1\frac{kJ}{mol})$$

$$CCl_4 + 2\,H_2O \rightarrow CO_2 + 4\,HCl\;(\Delta H_{773K} = -185.6\frac{kJ}{mol})$$

Oxidation of chloromethanes:

$$CH_3Cl + O_2 \rightarrow CO + HCl + H_2O\;(\Delta H_{773K} = \;-359.3\frac{kJ}{mol})$$

$$2\,CH_2Cl_2 + O_2 \rightarrow 2\,CO + 4\,HCl\;(\Delta H_{773K} = \;-393.7\frac{kJ}{mol})$$

$$2\,CHCl_3 + O_2 \rightarrow 2\,CO + 2\,HCl + 2\,Cl_2\;(\Delta H_{773K} = \;-203.4\frac{kJ}{mol})$$

$$CCl_4 + O_2 \rightarrow CO_2 + 2\,Cl_2\;(\Delta H_{773K} = \;-302.8\frac{kJ}{mol})$$

3. Experimental Definitions and Calculations

3.1 Thermometric Performance and Temperature Determination

The Boltzmann equation

$$(Eq.S1)\frac{I_{5D1}}{I_{5D0}} = A * e^{\frac{-\Delta E}{k_B T}}$$

where I is the $\frac{I_{5D1}}{I_{5D0}}$ is the intensity ratio of the two thermally coupled states, here the $^5D_1$ and 5D_0 of Eu^{3+}, and A is a constant, is applied to calculate the energy gap between the thermally coupled states for calibration measurements and for the determination of the catalyst temperature. When Boltzmann thermometers are applied, the relative sensitivity of the thermometer can be expressed as[2]

$$(Eq.S2)\ S_R = \frac{\Delta E}{k_B T^2}\ (in\ \%\ K^{-1})$$

where ΔE is energy gap between the two thermally coupled states, k_B is the Boltzmann constant and T is the temperature in K. The temperature uncertainty can be expressed by[3]

$$(Eq.S3)\ \sigma_T = \frac{1}{S_R}\sqrt{\frac{1}{A} + \frac{1}{B}}\ (in\ K)$$

where A and B are the integrated count rates in the regions of interest for the $^5D_1$ and $^5D_0$ emission.

3.2 Data Analysis Procedure

The raw spectral data was converted before it was suited for thermometric applications. The first step was the dark subtraction to correct for stray light. Subsequently, any faulty measurements, i.e. measurements approaching the maximum count rate and low signal measurements (below 10% of the maximum count rate) that result in a large σ_T, were removed from the dataset. Next, the wavelength was converted to energy scale according to

$$(Eq.S4)\ E = \frac{1}{\lambda} * 10^{-7}\ (in\ cm^{-1}).$$

As the x-axis went from evenly spaced intervals to non-evenly spaced intervals, the spectral intensity has to be corrected accordingly.[4] The Jacobian transformation

$$(Eq.S5)\ I_{corrected}(\lambda) = \frac{I_{spectrum}(\lambda)}{E(\lambda)^2}$$

was applied where *I* and *E* are the intensity and energy at a specific wavelength. Next, the spectra were normalized to 100 and the regions of interest were fitted with multiple Lorentzians and a baseline, given by the basic function

$$(Eq.S6)\ I_{fit} = \sum_i \frac{a_i * {w_i}^2}{(E(\lambda) - c_i)^2 * {w_i}^2} + z$$

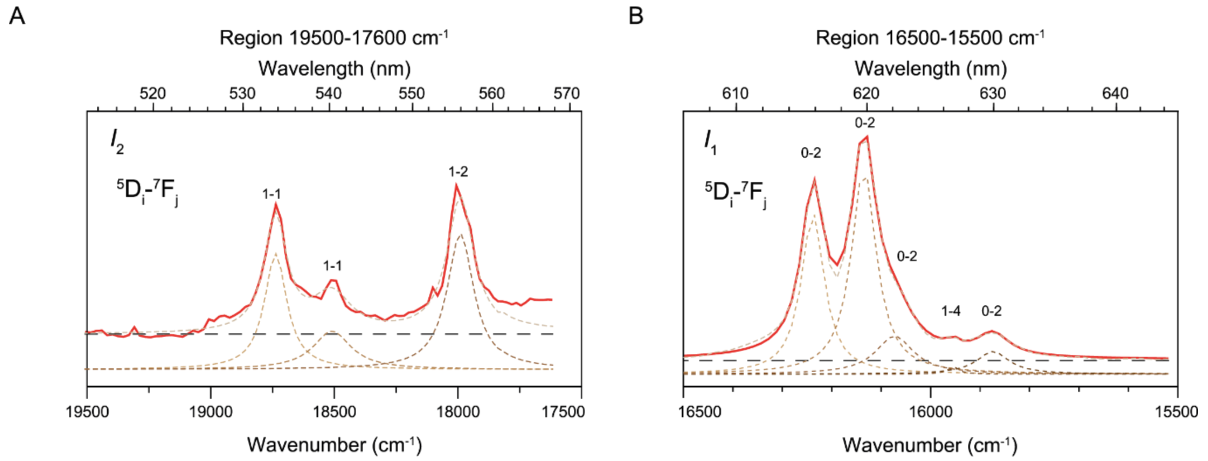

Figure S1. Regions of interest for (A) I_2 and (B) I_1. Three Lorentzians were used as input for I_2 and five Lorentzians were used to fit the I_1 region, of which only the $^5D_0 \rightarrow {}^7F_2$ were used as input for I_1.

where a, w, c and z are the peak amplitude, peak width, peak center and a constant (Figure S1). The area of the sum of the Lorentzian (without z) was applied as input for the Boltzmann equation (Eq.S1).

3.3 Heat Transfer Calculations By Convection and Radiation

Heat generated by the reaction (Q_r) was calculated according to

$$(Eq.S7)\ Q_r = XC_{CH_4}F_g\Delta H_r \approx 0.02W$$

with a methane conversion $X \approx 0.3$, inlet CH_4 concentration $C_{CH_4} = 0.1\frac{p}{RT} \approx 1.5$ mol m^{-3}, gas flow $F_g = 3 \cdot 10^{-7}$ m^{3}s^{-1} and reaction enthalpy $\Delta H_r = 158 \cdot 10^3$ J mol^{-1}. If all heat would be withdrawn by reaction gas mixture, the temperature increase of the gas (DT_g) can be calculated with

$$(\text{Eq. S8})\ \Delta T_g = \frac{Q_r}{F_g\rho_g Cp_g} = \frac{XC_{CH4}\Delta H_r}{\rho_g Cp_g} \approx 120K$$

with a gas density $\rho_g \approx 0.5$ kg m^{-3} and a heat capacity of the gas $Cp_g \approx 1150$ J kg^{-1} K^{-1}. As the inlet gas temperature is assumed to be equal to the oven temperature (Figure S5), convection by reaction mixture is presumably not the predominant cooling mechanism due to the large calculated DT_g. Therefore, Q_r needs to be withdrawn radially outwards by either conduction or radiation. The heat generated by reaction per sieved catalyst particle ($Q_{r,p}$) is calculated with

$$(Eq.S9)\ Q_{r,p} = \frac{Q_r}{n_p}.$$

Assuming spherical catalyst particles, the number of particles, n_p, is approximated by

$$(Eq.S10)\ n_p = \frac{V_{bed}(1-\epsilon_{bed})}{V_p} = \frac{V_{bed}(1-\epsilon_{bed})}{\frac{1}{6}\pi d_p^3}$$

with a particle diameter $d_p = 275 \cdot 10^{-6}$m, void factor $\epsilon_{bed} \approx 0.43$, bed volume $V_{bed} = 3.4 \cdot 10^{-7}\ m^3$ and the number of particles $n_p \approx 17700$, resulting in $Q_{r,p} = 1.2 \cdot 10^{-6}$ W. At 773K (500 °C), the particle emission $Q_{e,p}$ can be calculated according to

$$(Eq.S11)\ Q_{e,p} = \sigma T^4 \pi d_p^2 = 4.8 \cdot 10^{-3}\ W$$

where s is the Stefan-Boltzmann constant (s = 5.6703*10^{-8} W m^{-2} K^{-4}). Even at extremely low values of material emissivity, particle emission $Q_{e,p}$ is at least 2 orders of magnitude larger than the heat generated per particle $Q_{r,p}$. Hence, any increase in particle temperature due to reaction would immediately be lost by radiation to the environment. Therefore, the

temperature is likely to be uniform throughout the bed. At the walls of the reactor, heat is lost to the environment (i.e. the oven), most likely by radiation as well as

$$(Eq. S12)\ Q_{e,reactor} = \sigma T^4 A = 6.7W$$

with the outer reactor surface $A = 3.3 \cdot 10^{-4}$ m^2 and $T = 773K$. Again, $Q_{e,reactor}$ is more than 2 orders of magnitude larger then Q_r. Hence, any temperature increase due to reaction will quickly be emitted by radiation, until equilibrium at oven temperature is reached. It can therefore be concluded that the bed temperature most likely is constant throughout its volume, at oven temperature. Still, heat is generated by the reaction and the catalyst bed temperature must be higher than the surrounding

$$(Eq. S13)\ Q_{e,reactor} - Q_{e,0} = Q_r = \epsilon_m \sigma A (T^4 - T_0^4)$$

with $Q_{e,0}$ the total mission without reaction. However, the material emissivity of EuOCl is unknown and determination is outside the scope of this work. Nevertheless, assuming a particle emissivity ϵ_m of 0.1 would result in a ΔT of 5K, which is in the right order of magnitude. The calculations enabled us to explain the trends qualitatively but a more detailed model and more carefully controlled experiments are needed for the quantitative description of the temperature increase.

4. Additional Experimental Data

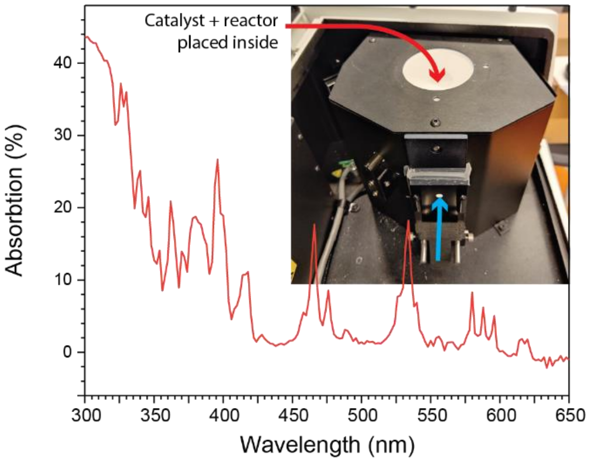

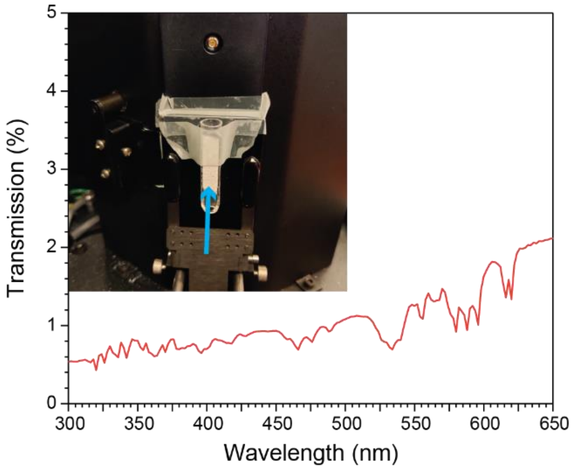

Figure S2. (A) Absorbance of a quartz reactor filled with EuOCl (125-425 μm sieve fraction) is measured by placed in the filled reactor in the integrating sphere. The beam (blue arrow) enters the pinhole and hits the filled reactor. Due to the geometry of the integrating sphere, scattered light has a high chance of hitting the sample again. Hence, the indicated absorption is not representative for a single absorption event, but gives a relative measure of the absorption at a certain wavelength. (B) Transmittance of the sample is measured through the filled quartz reactor. The quartz reactor with catalyst is placed in front of the pinhole and the beam passes through. Light enters the integrating sphere, where it is detected. The transmittance of the sample is quantitative and below 1 % at the laser wavelength of 375nm, which is a quantitative measure for the light transmitting through the catalyst bed and reactor.

A

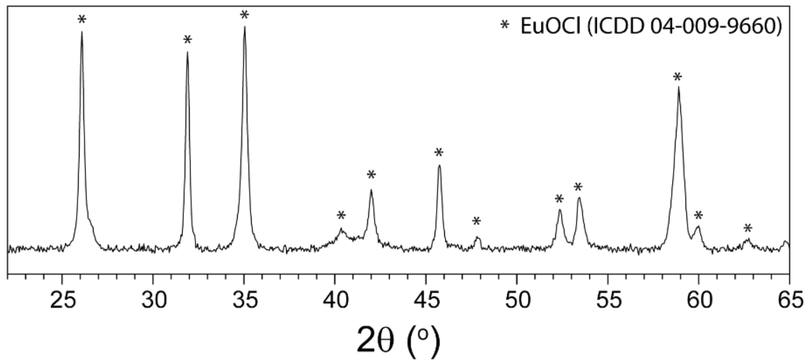

B

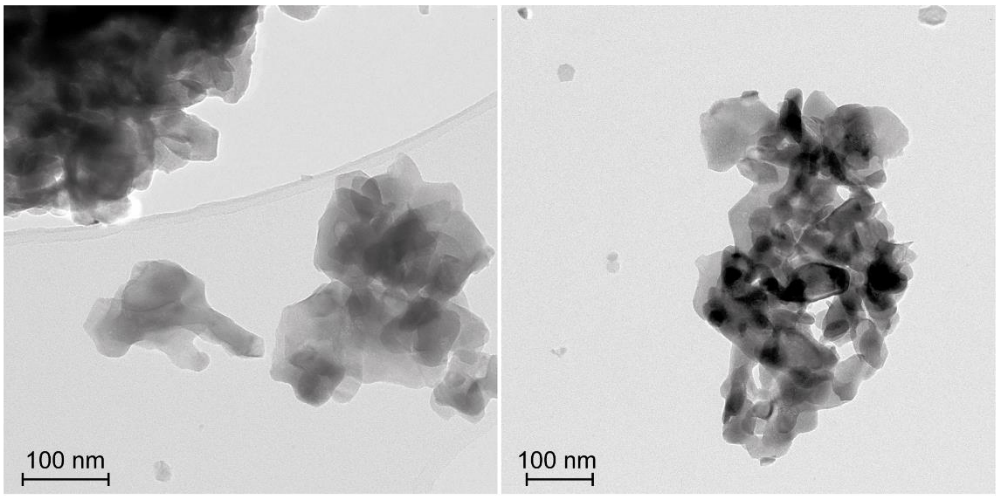

Figure S3. (A) X-ray diffractogram (XRD) performed on the as-synthesized EuOCl. All diffractions could be assigned to the reference EuOCl (ICDD 04-009-9660). (B) Transmission electron microscopy (TEM) images of as-synthesized EuOCl. Ill-defined particles of ten to hundreds of nanometers are formed which form larger agglomerates.

A

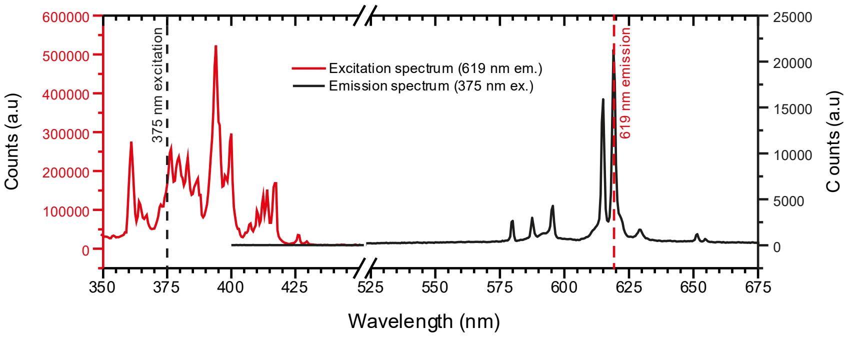

B

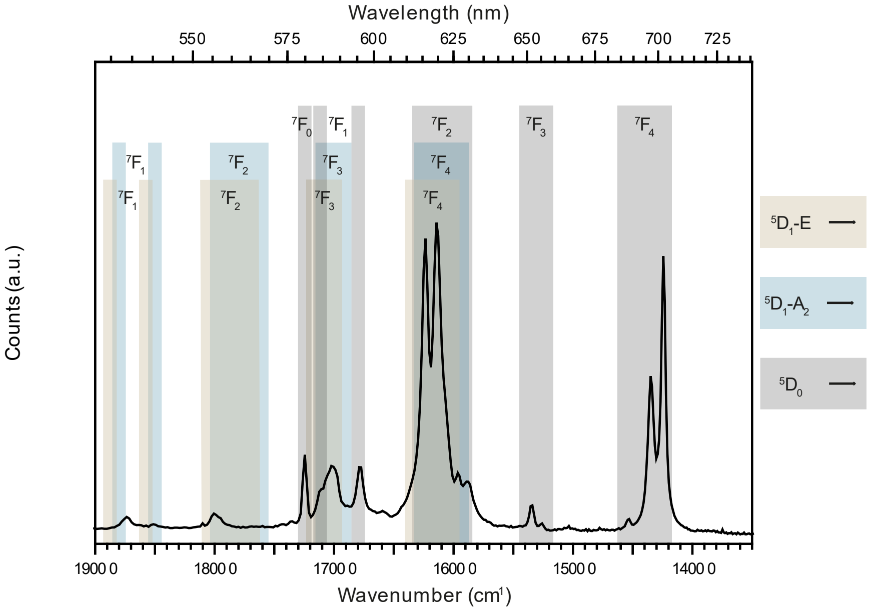

Figure S4. (A) Excitation (emission recorded at 619 nm) and emission (excited at 375 nm) spectrum of EuOCl at room temperature.[5] From the excitation spectrum, it becomes apparent that Eu^{3+} can be excited in the intra 4-F transition at 375nm. The emission spectrum clearly yields the Eu^{3+} emission spectrum. (B) Emission spectrum of EuOCl at 500 °C excited at 375nm where the Eu^{3+} emission are labelled according to the energy diagram of Eu^{3+} (Figure 1A).[6]

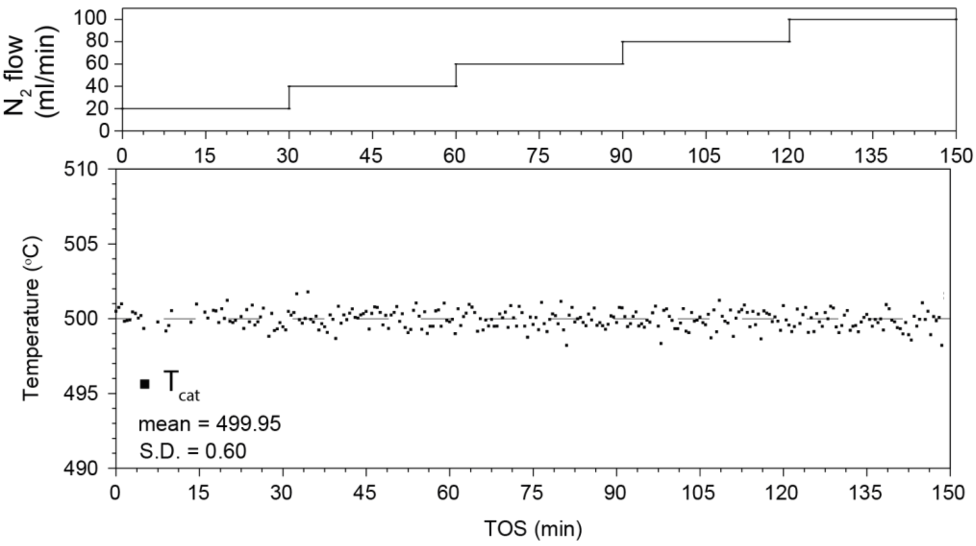

Figure S5. The catalyst temperature as determined by the Boltzmann model (T_{cat}) is plotted versus the time-on-stream (TOS) at an oven temperature (T_{oven}) of 500 °C. No cooling of the catalyst with increasing gas flow (20 - 100 mL/min N_2) was observed, indicating that the inlet gas was already heated to the T_{oven} before contact with the catalyst. The standard deviation (S.D.) of 0.6 °C is about twice the temperature uncertainty σ_T. The S.D. provides a good indication of the accuracy of the temperature measurement.

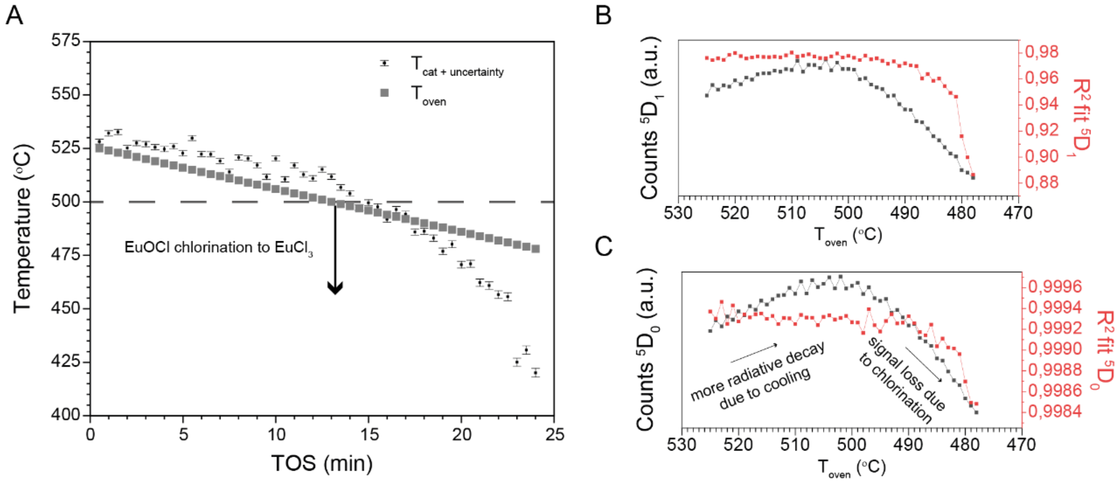

Figure S6. (A) The catalyst temperature as determined by the Boltzmann model (T_{cat}) and the oven temperature (T_{oven}) plotted versus the time-on-stream (TOS). The T_{oven} was gradually decreased by 2 °C/min from 525 °C under 50 % HCl/He (20 ml/min total flow). From 0 - 12 min TOS, the T_{cat} followed the T_{oven} closely. After 12 min TOS, the apparent T_{cat} dropped below the T_{oven} due to the larger uncertainty in the temperature measurement and the changing emission spectrum. The fitted (B) $^5D_1$ and (C) $^5D_0$ revealed that at 500 °C, a maximum in the counts was obtained, after which it rapidly decreased at lower temperatures. Chlorination of the catalyst at a temperature below 500 °C quenched the luminescence signal, reducing the counts. The decrease in counts was also reflected in the R^2 of the fits, which decrease rapidly, especially at temperatures lower than 490 °C.

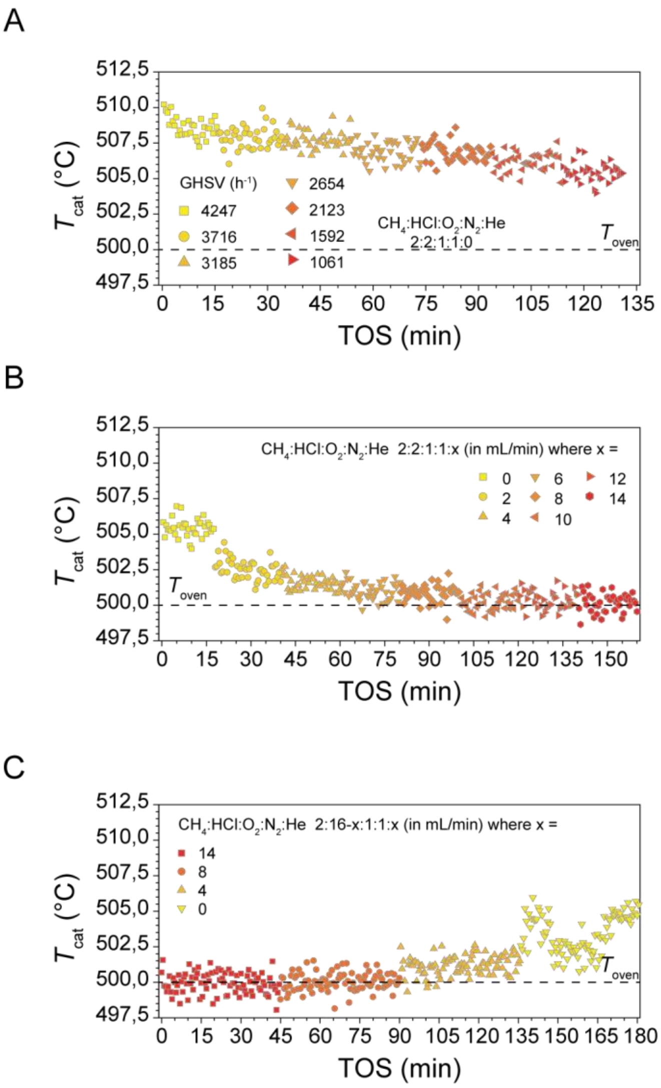

Figure S7. Three different variations in feed compositions were tested at T_{oven} = 500 °C and the influence on the catalyst temperature as determined by the Boltzmann model (T_{cat}) is plotted versus the time-on-stream (TOS). (A) The gas hourly space velocity (GHSV) was varied while keeping the feed composition unchanged (CH_4:HCl:O_2:N_2:He 2:2:1:1:0, total flow varying from 6 to 24 mL/min). (B) The feed was diluted with inert gas, decreasing the catalyst temperature (CH_4:HCl:O_2:N_2:He 2:2:1:1:x (in mL/min) where x = 0 – 14 with increments of 2). (C) The HCl:He ratio was varied at a constant GHSV (CH_4:HCl:O_2:N_2:He 2:16-x:1:1:x (in mL/min) where x = 0, 4, 8, 14). Reaction conditions: T_{oven} = 500 °C, W_{cat} = 500 mg, V_{cat} = 0.34 cm^3.

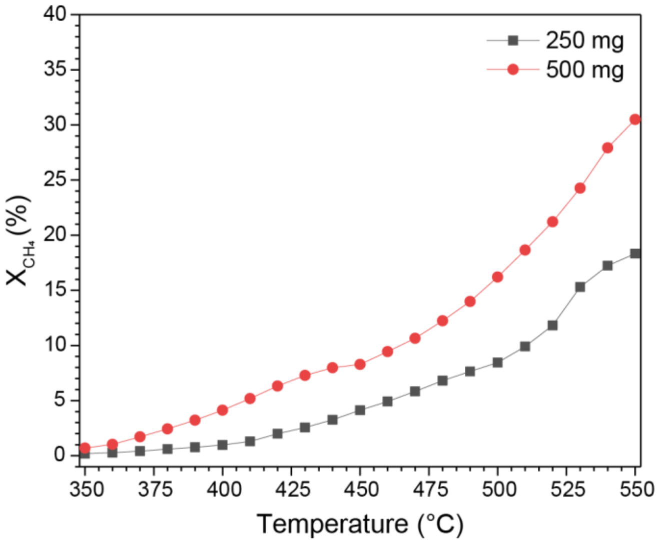

Figure S8. Methane conversion (X_{CH4}) plotted versus the temperature for EuOCl tested with different weight loadings of catalyst. The activity of the 500 mg is roughly double that of the 250 mg weight loading. Reaction conditions: T_{oven} = 350-550 °C with steps of 10 °C, stabilized for 45 min, CH_4:HCl:O_2:N_2:He 2:2:1:1:14 (in mL/min).

5. References

[1] B. Terlingen, R. Oord, M. Ahr, E. Hutter, C. van Lare, B. M. Weckhuysen, *ACS Catal.* **2021**, *11*, 10574–10588.

[2] X. Wang, Q. Liu, Y. Bu, C. S. Liu, T. Liu, X. Yan, *RSC Adv.* **2015**, *5*, 86219–86236.

[3] T. P. van Swieten, A. Meijerink, F. T. Rabouw, *ACS Photonics* **2022**, *9*, 1366–1374.

[4] J. Mooney, P. Kambhampati, *J. Phys. Chem. Lett.* **2013**, *4*, 3316–3318.

[5] D. Kim, J. R. Jeong, Y. Jang, J. S. Bae, I. Chung, R. Liang, D. K. Seo, S. J. Kim, J. C. Park, *Phys. Chem. Chem. Phys.* **2019**, *21*, 1737–1749.

[6] L. G. Deshazer, G. H. Dieke, *J. Chem. Phys.* **1963**, *38*, 2190–2199.
